# Supplementary material for: Ion channel nano-diagnostics for ER+ breast cancer
Source: J Biol Chem. 2026 Oct 8;302(12):113623. doi: 10.1016/j.jbc.2026.113623 (PMC13760947; doi:10.1016/j.jbc.2026.113623)
Supplement: Supplementary file 1 [file mmc1.pdf]

Supporting Information

Ion Channel Nano-Diagnostics for ER+ Breast Cancer

Manos Gkikas[*,#,&] *Evangelos Dadiotis,*[#] *Mehreen Zaka,*[#] *Nihal Aly,*[#,^] *Kim Chan,*[#] *Diomedes. E. Logothetis*[*,#,&]

[#] Northeastern University, Department of Pharmaceutical Sciences, Boston, Massachusetts 02115, U.S.A.

[^] Alexandria University, Department of Botany and Microbiology, Alexandria, Egypt.

[&] Northeastern University, Center for Drug Discovery, Boston, Massachusetts 02115, U.S.A.

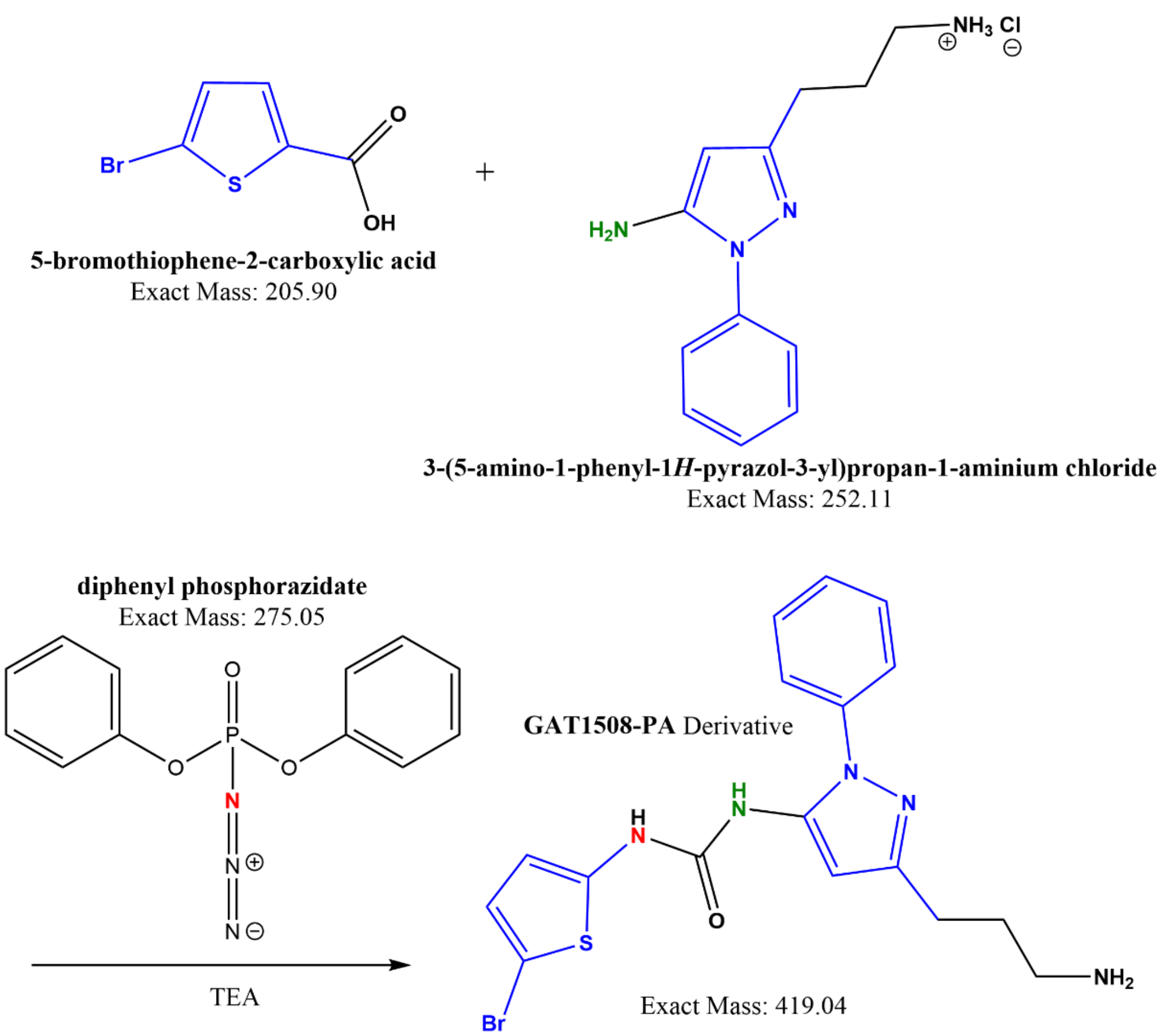

Scheme S1. Reaction scheme between 5-bromothiophene-2-carboxylic acid and benzyl pyrazole alkylated amines to produce alkylamine derivatives of GAT1508 in the presence of TEA and DPPA.

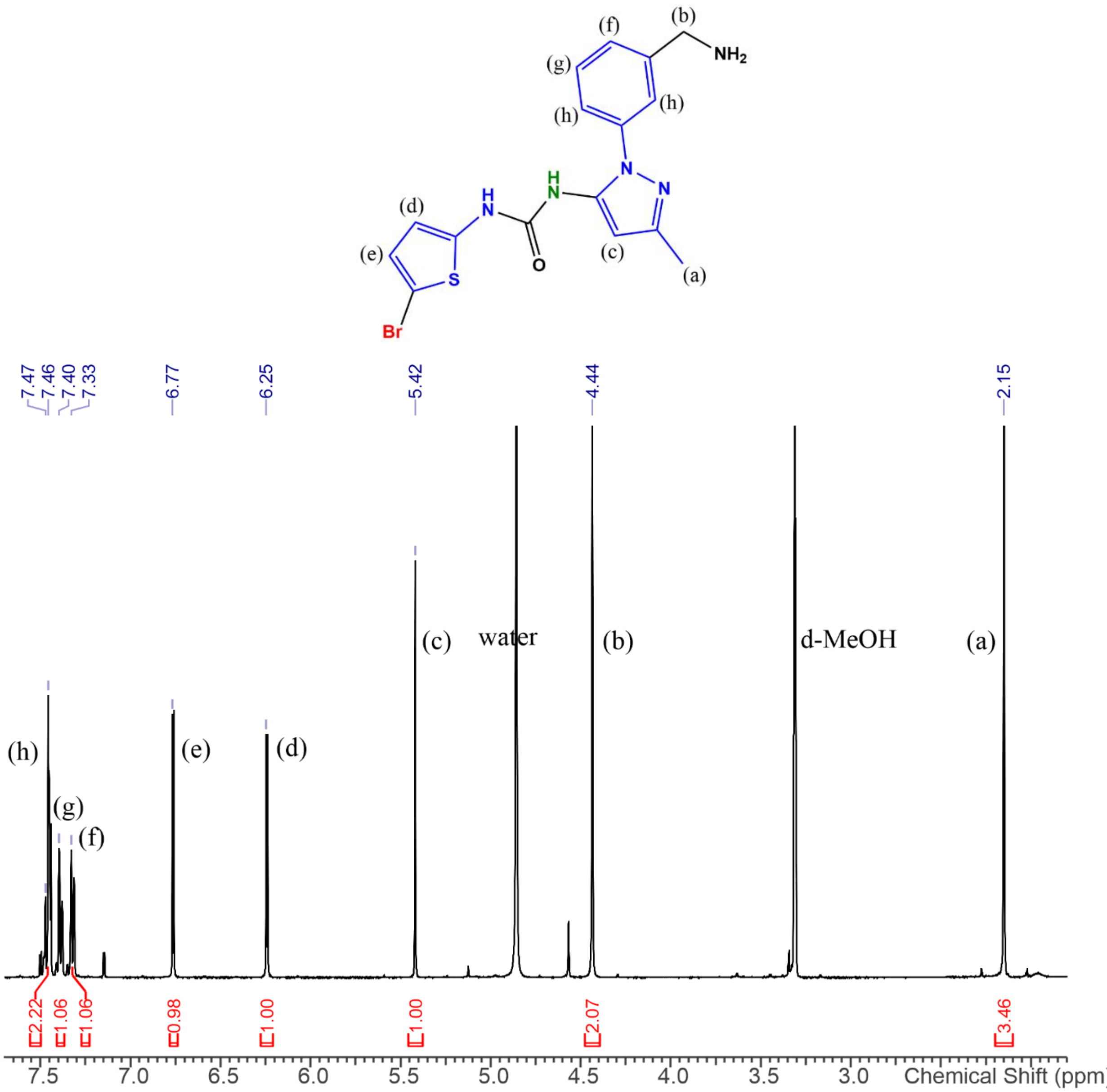

Figure S1. NMR of GAT1508 ethylamine in d-MeOH. The urea protons rapidly exchange with d-MeOH and thus cannot be seen.

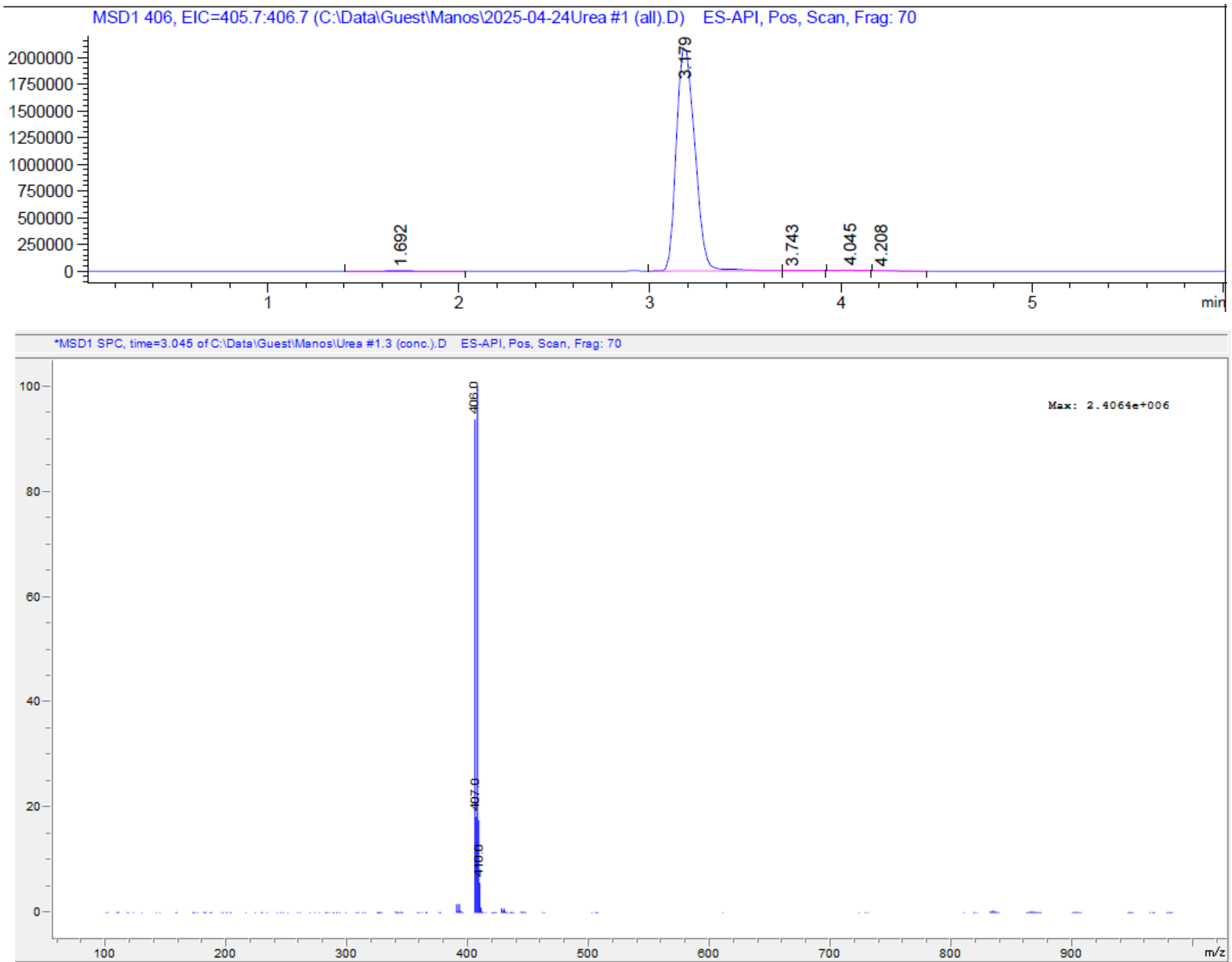

Figure S2. MS of GAT1508 ethylamine with a molecular weight of 405 g/mol.

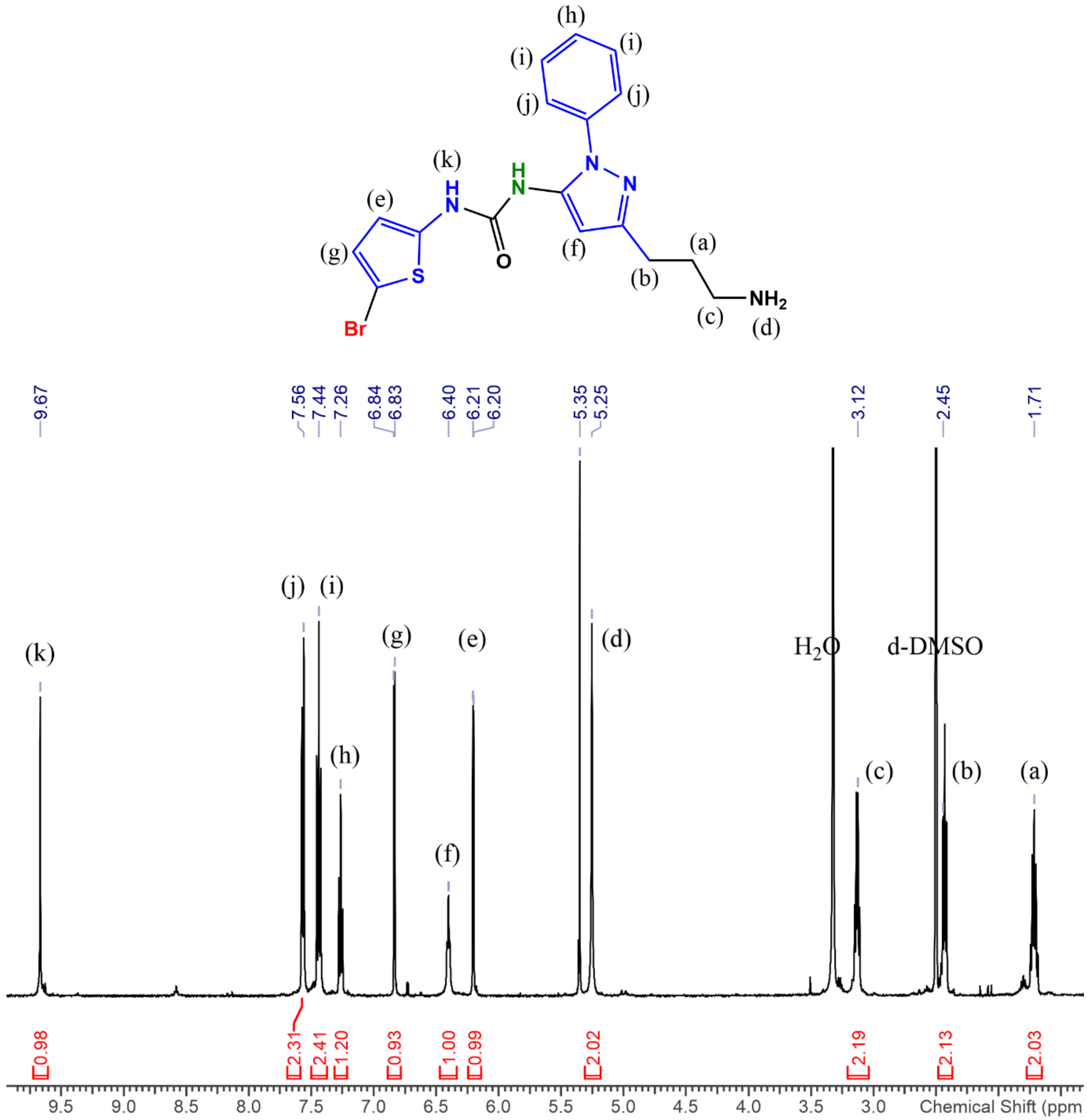

Figure S3. NMR of GAT1508 propylamine in d-DMSO. Only one of the urea protons can be seen. This is reported in the literature with high hydrophilic compounds.

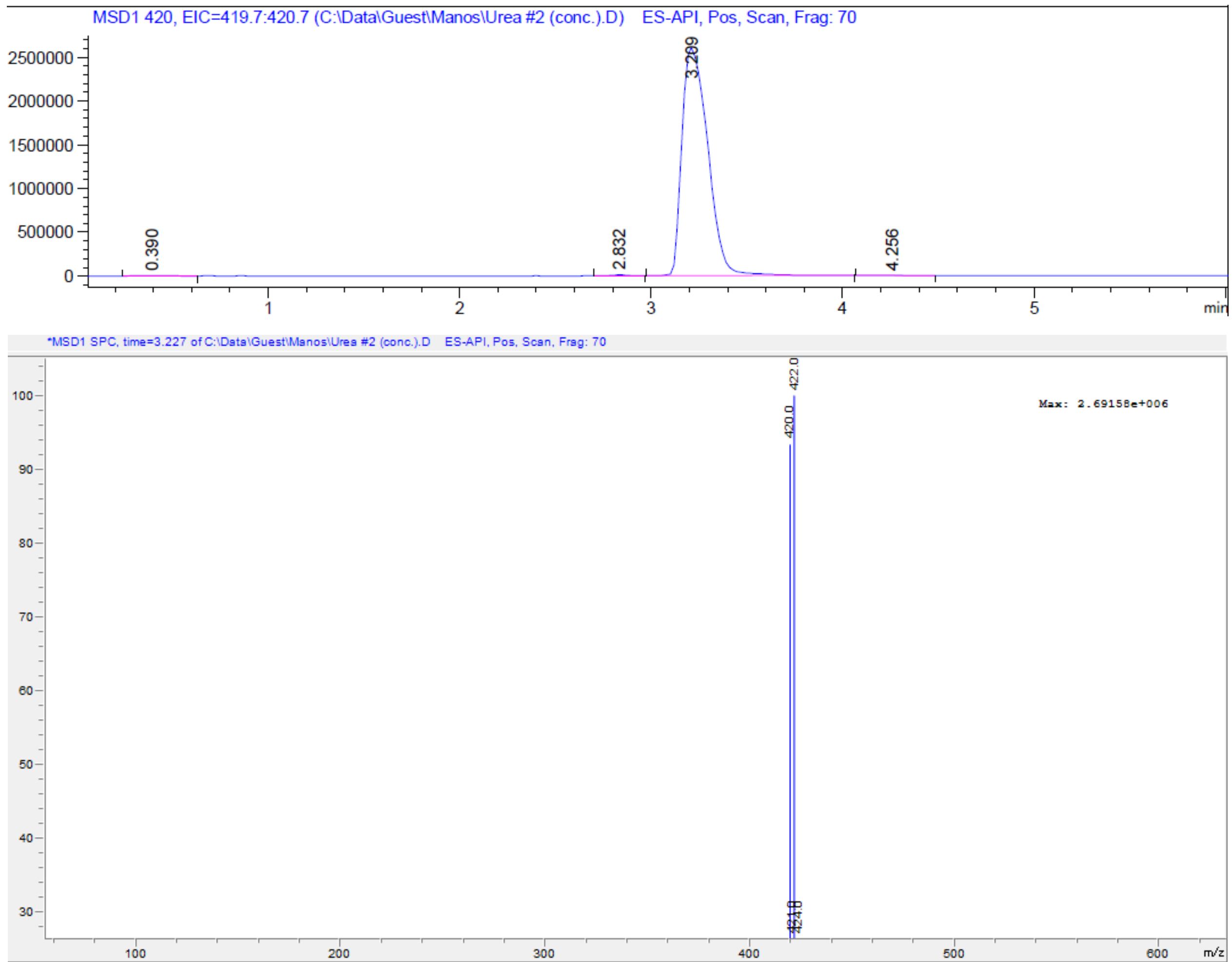

Figure S4. MS of purified GAT1508 propylamine with a molecular weight 419 g/mol.

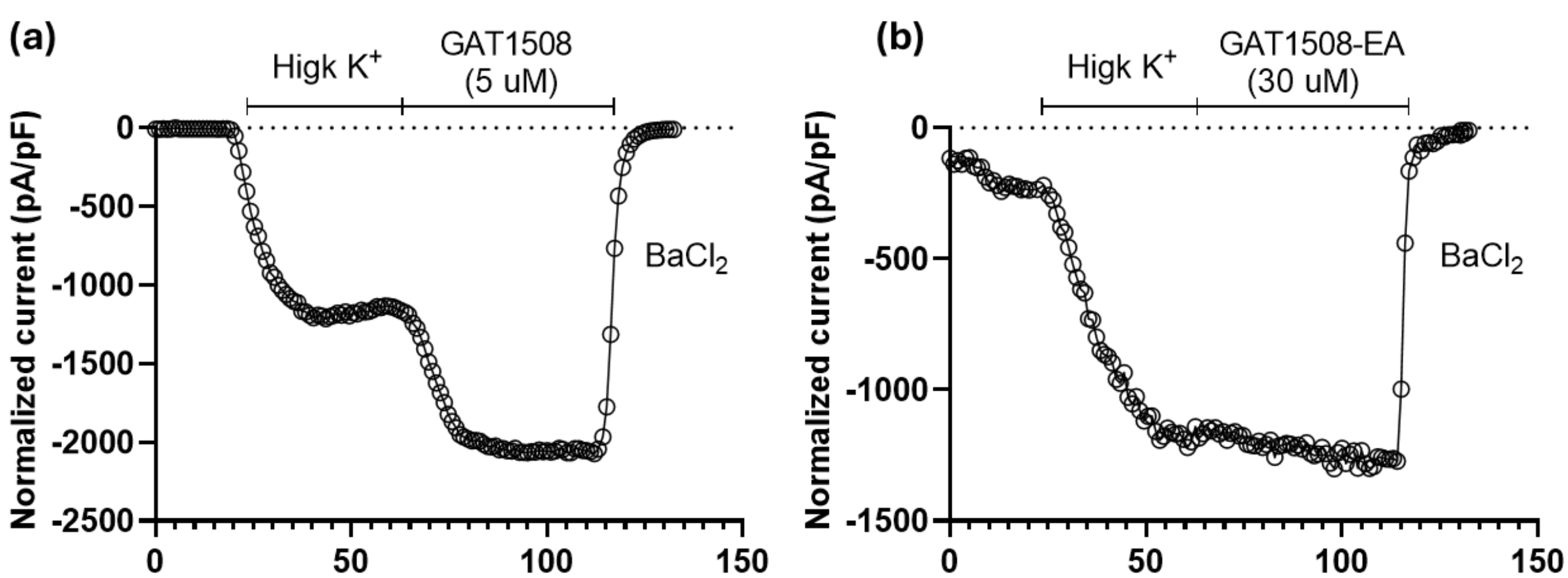

Figure S5. (a) Whole-cell patch-clamp recording of GAT1508 application on GIRK1/2 channels overexpressed in HEK293 cells. (b) Whole-cell patch-clamp recording of GAT1508-EA showing absence of interaction with GIRK1/2 channels overexpressed in HEK293 cells, with no detectable modulation of current amplitude under the tested conditions.

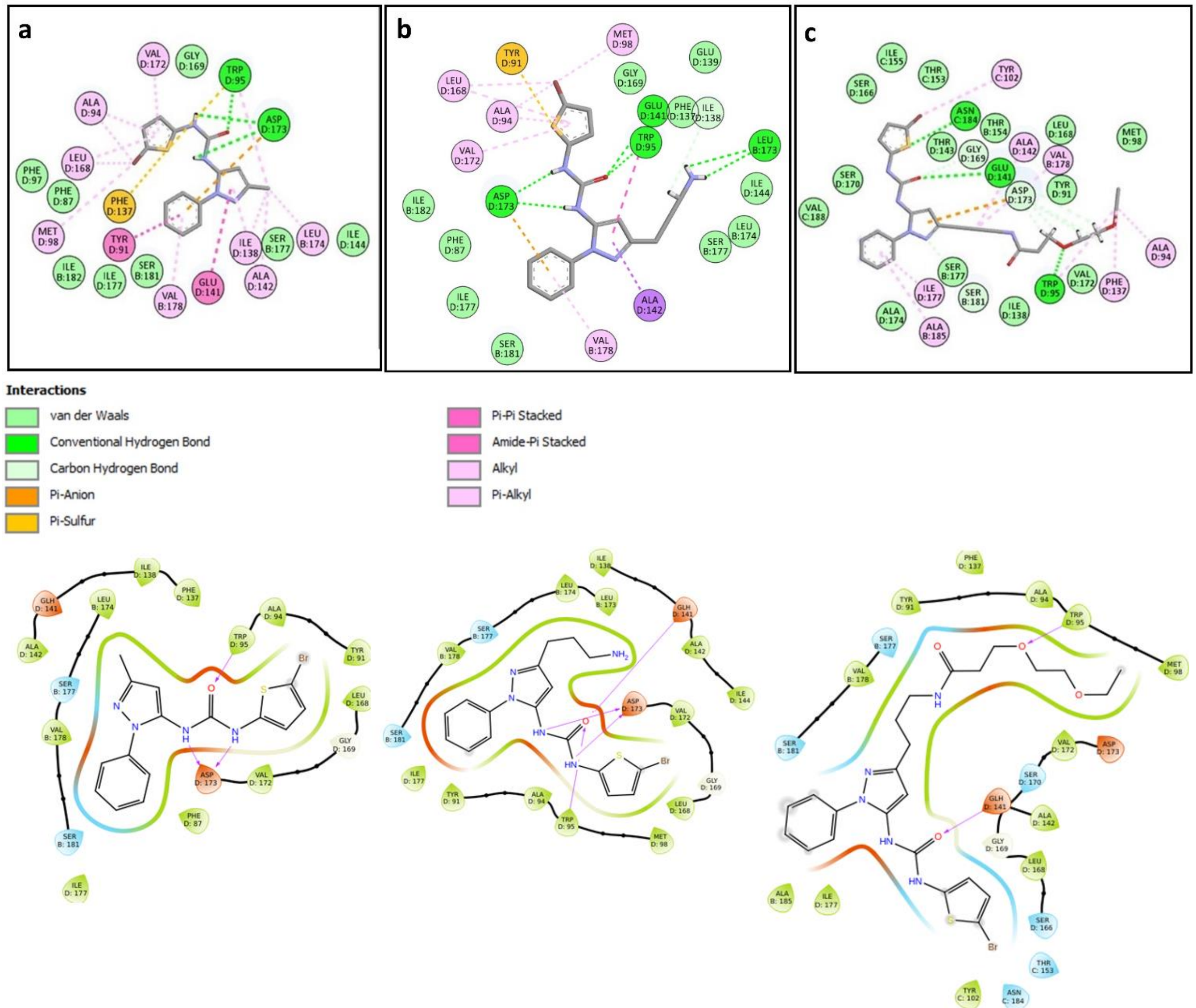

Figure S6. 2D interaction diagram of a) GAT1508, b) GAT1508-PA, and c) GAT1508-PA-EG2.

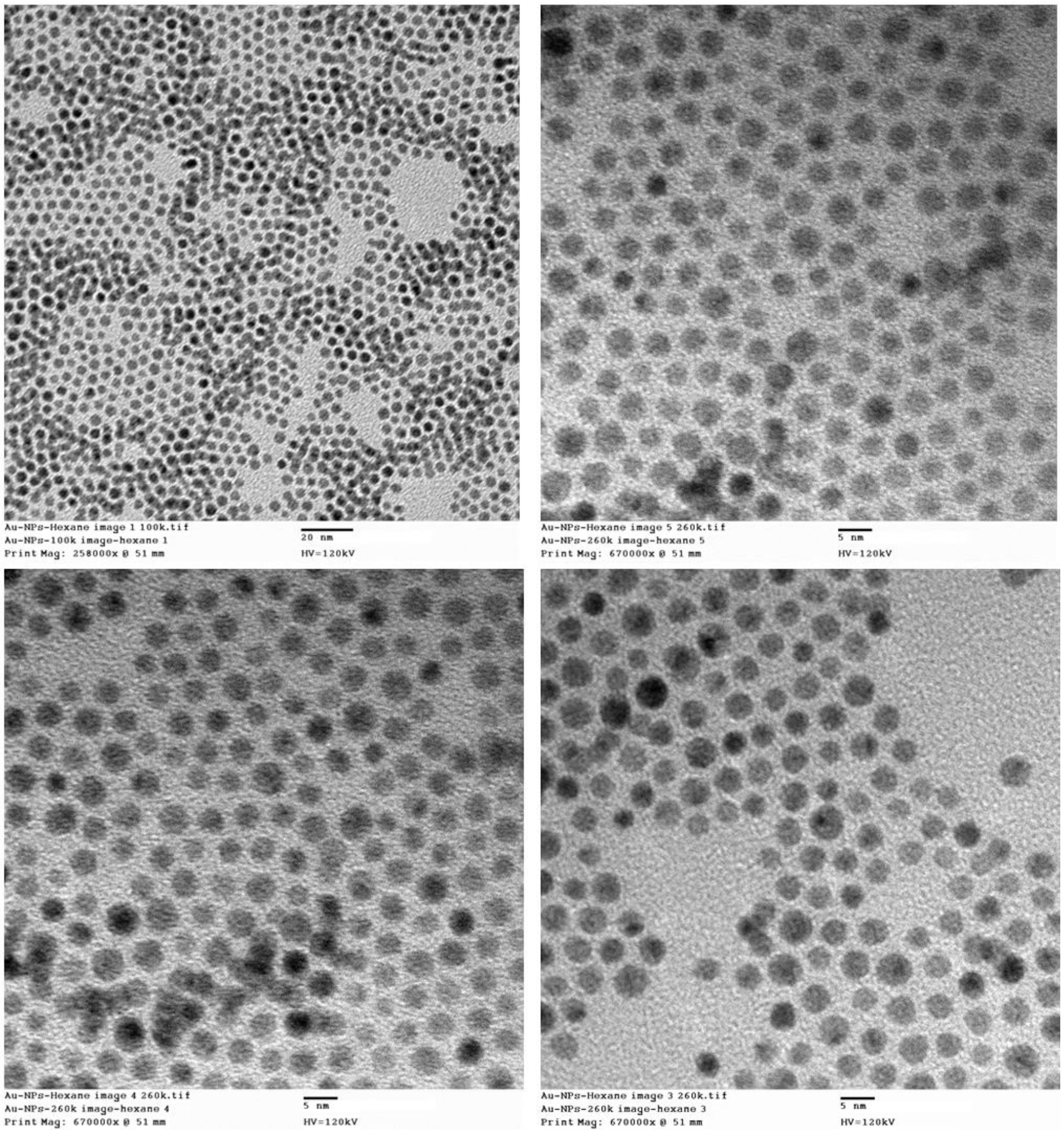

Figure S7. TEM of ~3.7 nm **dodecathiol-coated AuNPs** at 0.5 mg/mL in hexane.

Cumulative **TGA Table** for **dodecathiol-coated AuNPs**

**Sample**	**Decomp. T1 (°C)**	**Weight Loss 1 (%)**	**Decomp. T2 (°C)**	**Weight Loss 2 (%)**
1	307.3	11.4	-	-
2	296.9	11.7	-	-
3	299.6	12.0	-	-
Ave	**301** ± 5	**11.7** ± 0.3	-	-

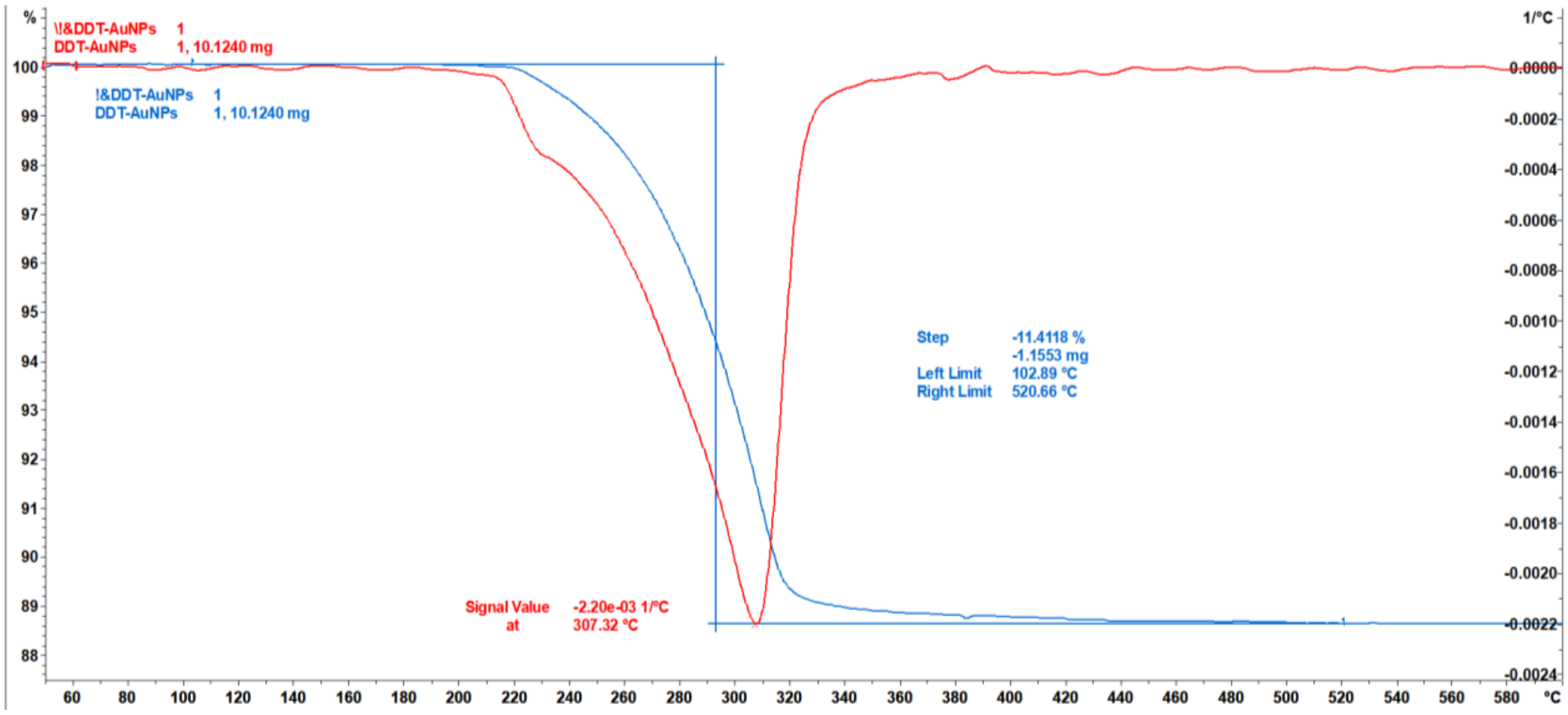

TGA of C12-S-AuNPs #1

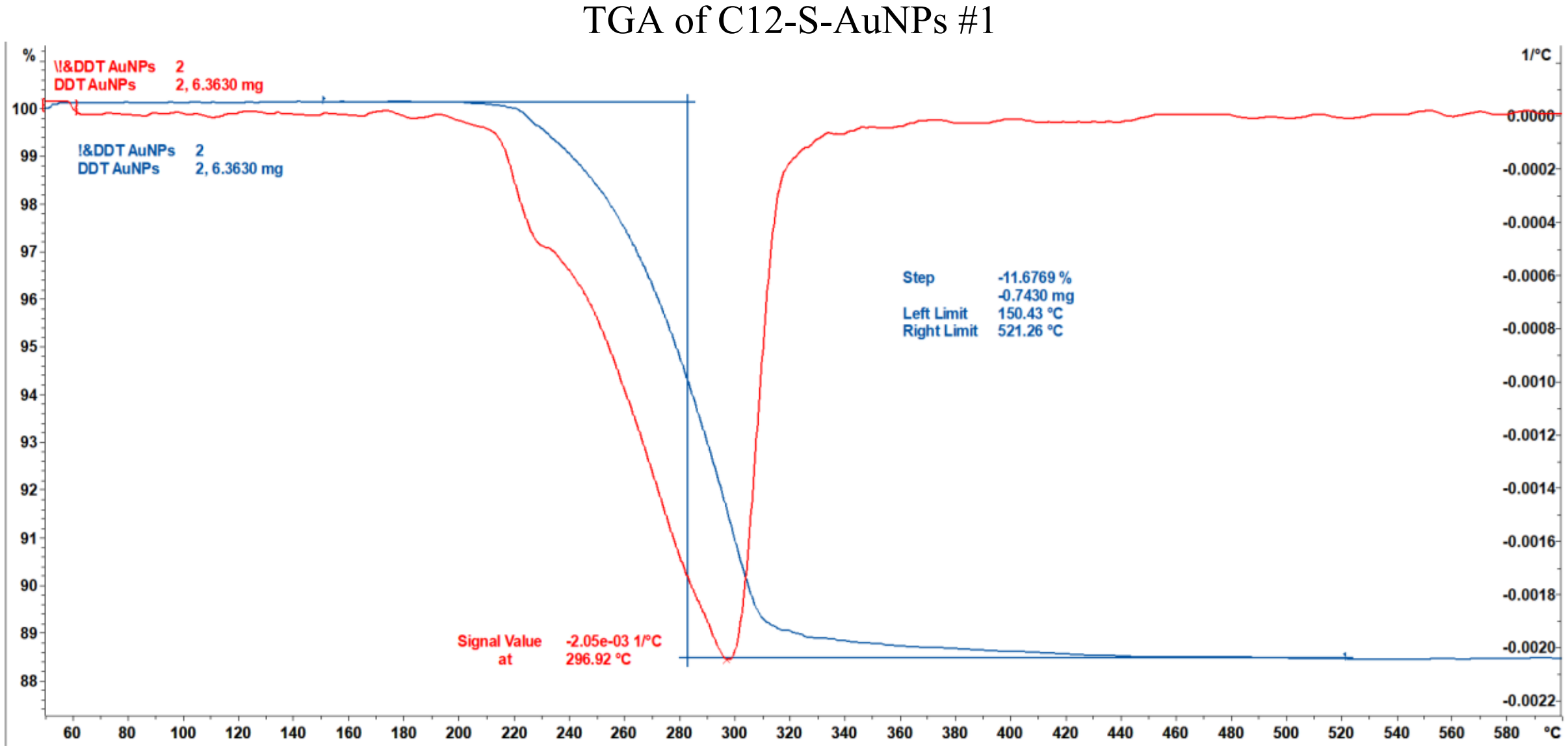

TGA of C12-S-AuNPs #2

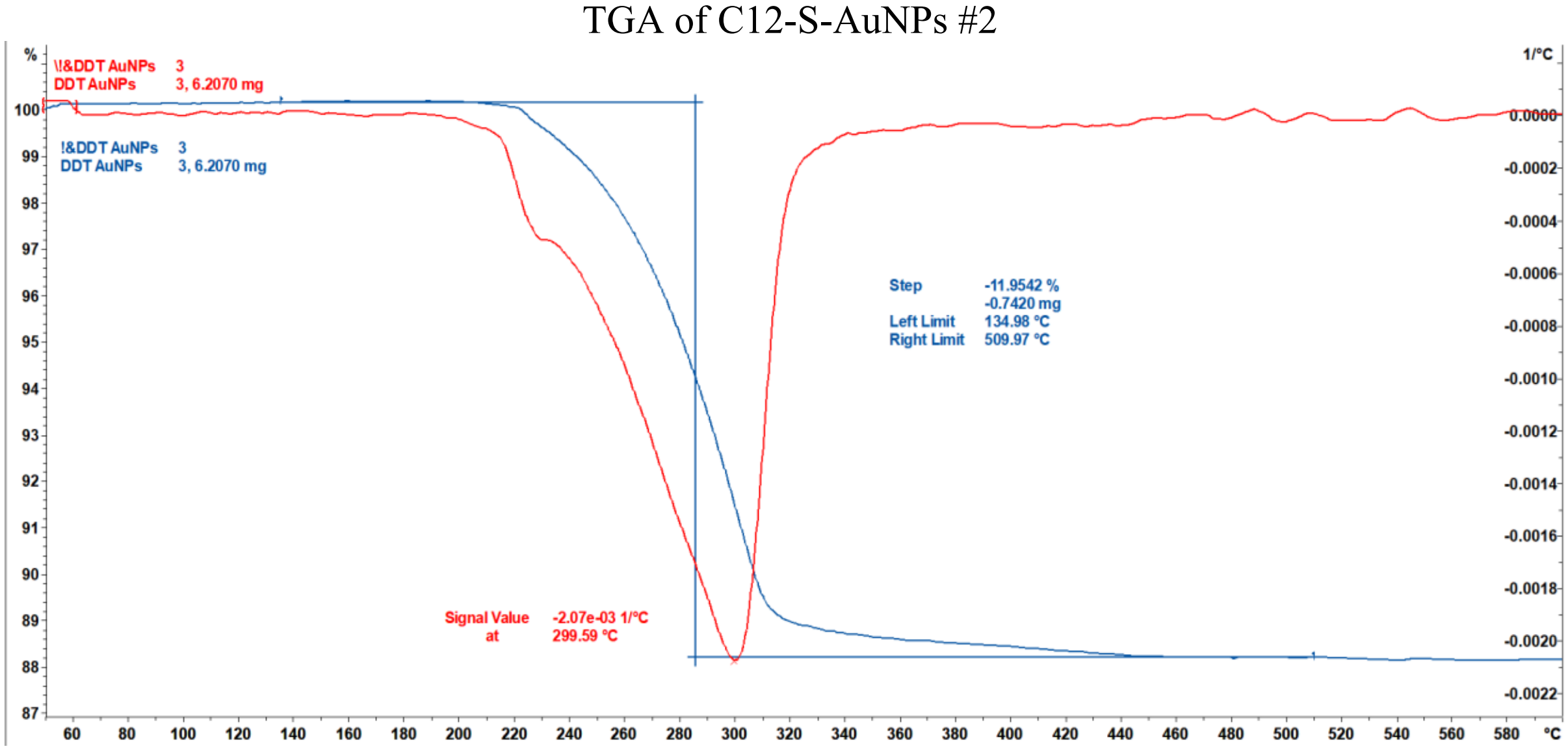

TGA of C12-S-AuNPs #3

Figure S8. TGA graphs of dodecathiol-coated AuNPs.

Cumulative **TGA Table** for **HOOC-PEG-SH**

Sample	Decomp. T1 (ºC)	Weight Loss 1 (%)	Decomp. T2 (ºC)	Weight Loss 2 (%)
1	-	-	416.1	96.8
2	-	-	415.8	96.6
3	-	-	415.2	96.4
Ave	-	-	**416** ± 1	**96.6** ± 0.2

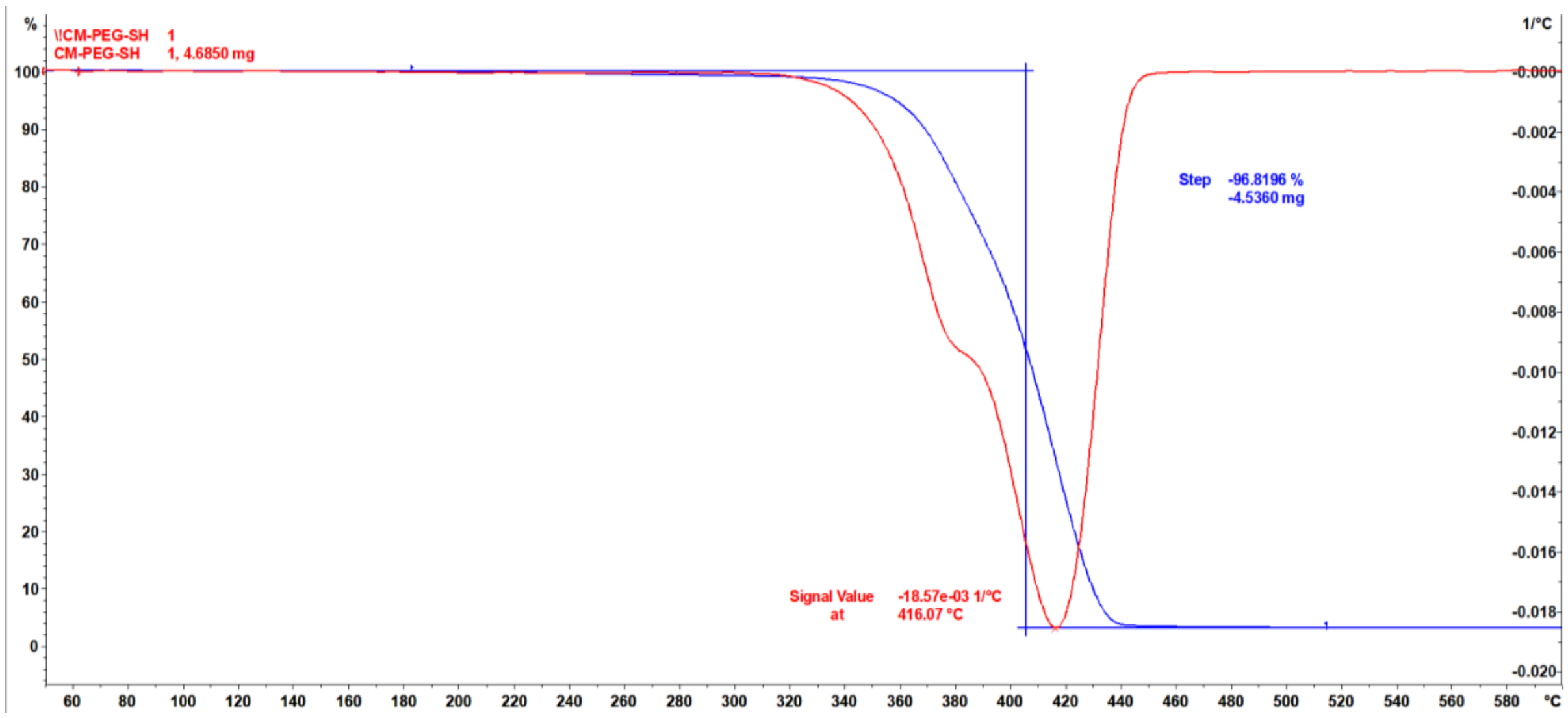

TGA of HOOC-PEG-SH #1

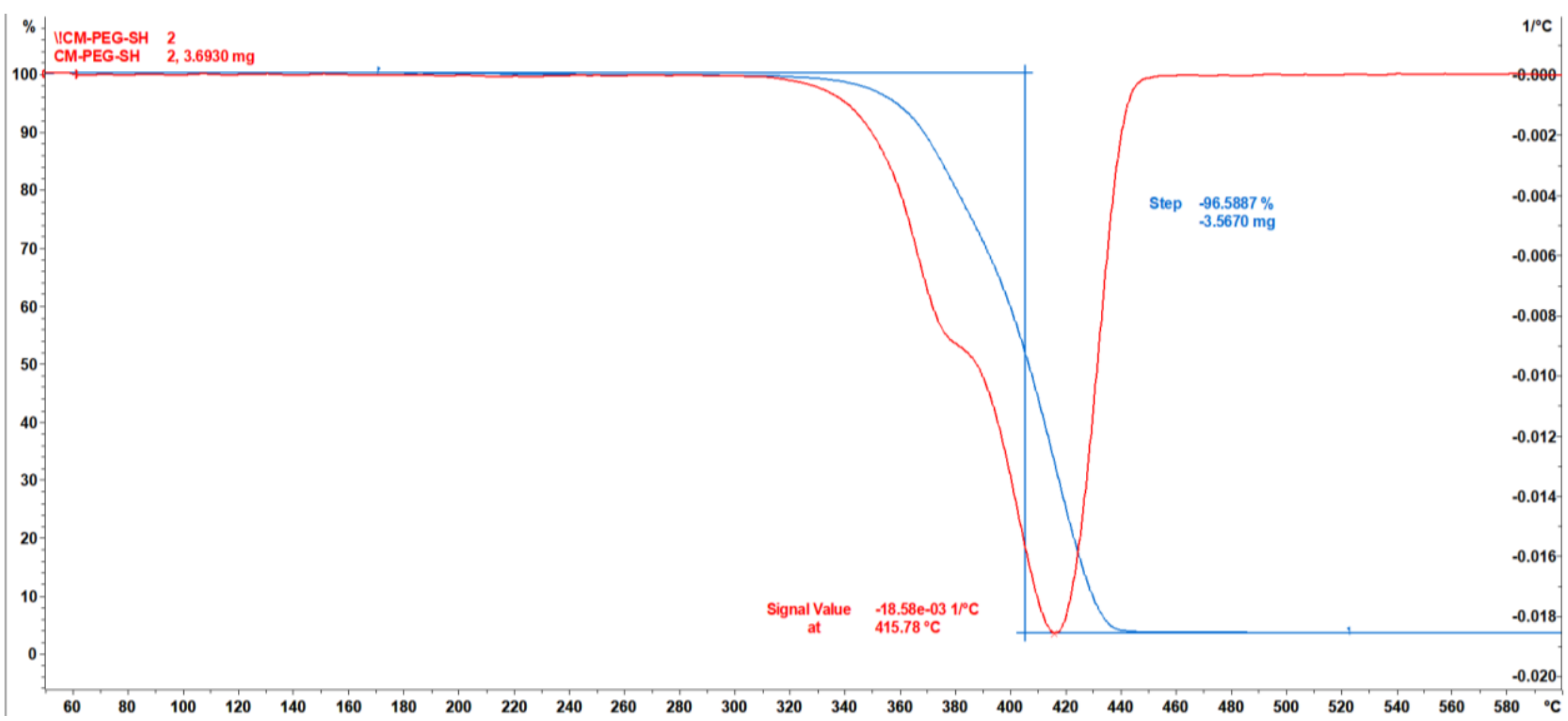

TGA of HOOC-PEG-SH #2

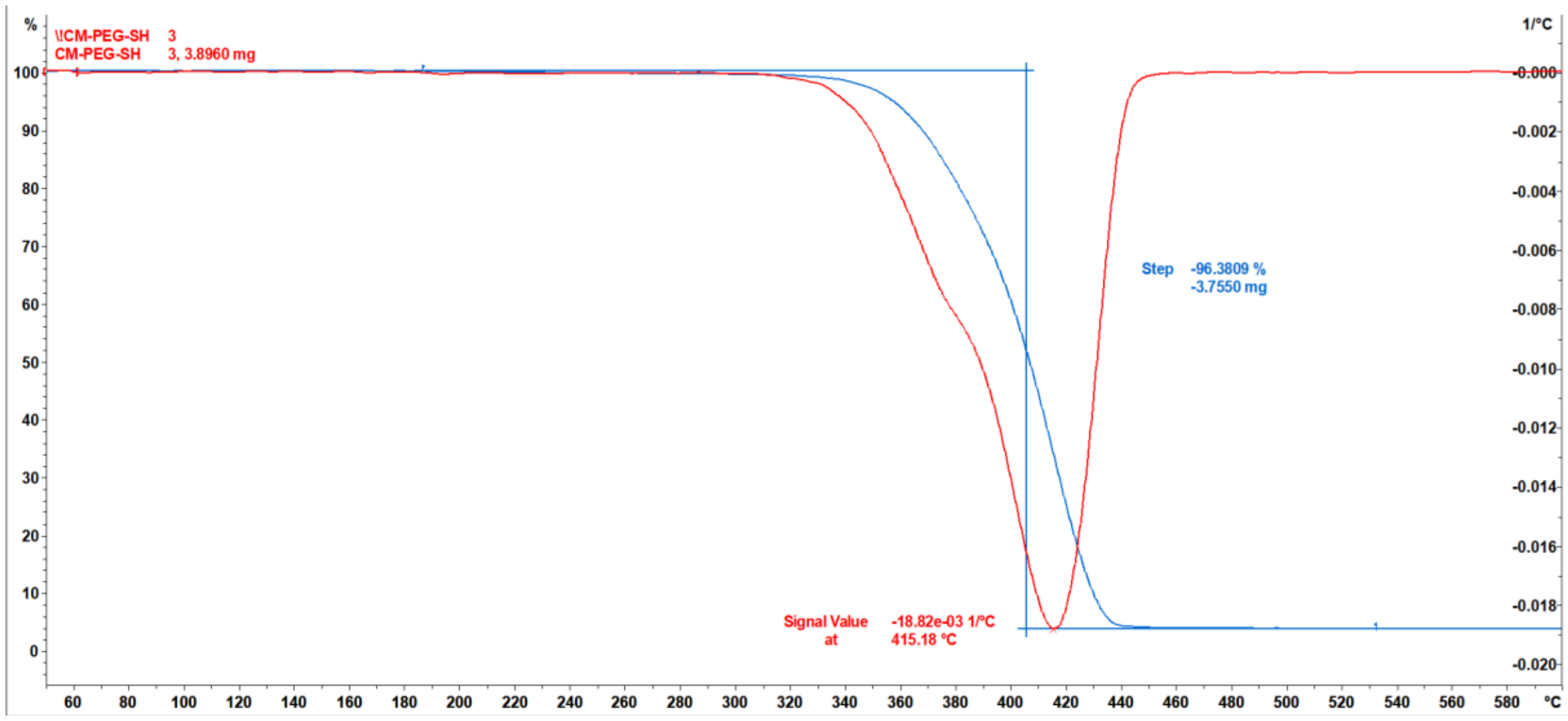

TGA of HOOC-PEG-SH #3

Figure S9. TGA graphs of **HOOC-PEG-SH**.

TGA Table of *purified* HOOC-PEG-AuNPs

Sample	Decomp. T1 (ºC)	Weight Loss 1 (%)	Decomp. T2 (ºC)	Weight Loss 2 (%)
1	277.9	5.9	404.5	33.6
2	282.4	6.2	400.6	35.1
3	276.1	5.8	398.8	34.8
Ave	**279** ± 3	**6.0** ± 0.2	**401** ± 3	**34.5** ± 0.8

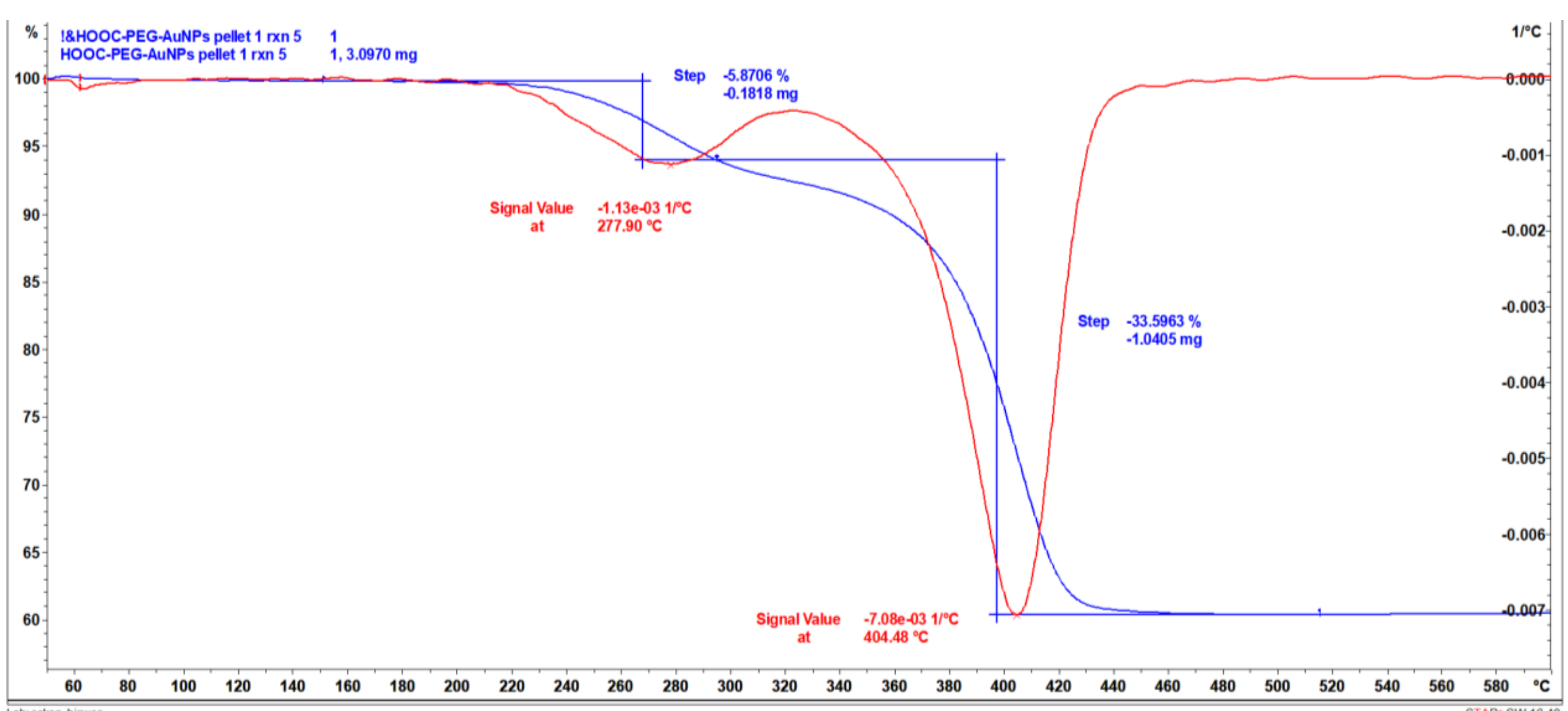

HOOC-PEG-AuNPs pellet #1

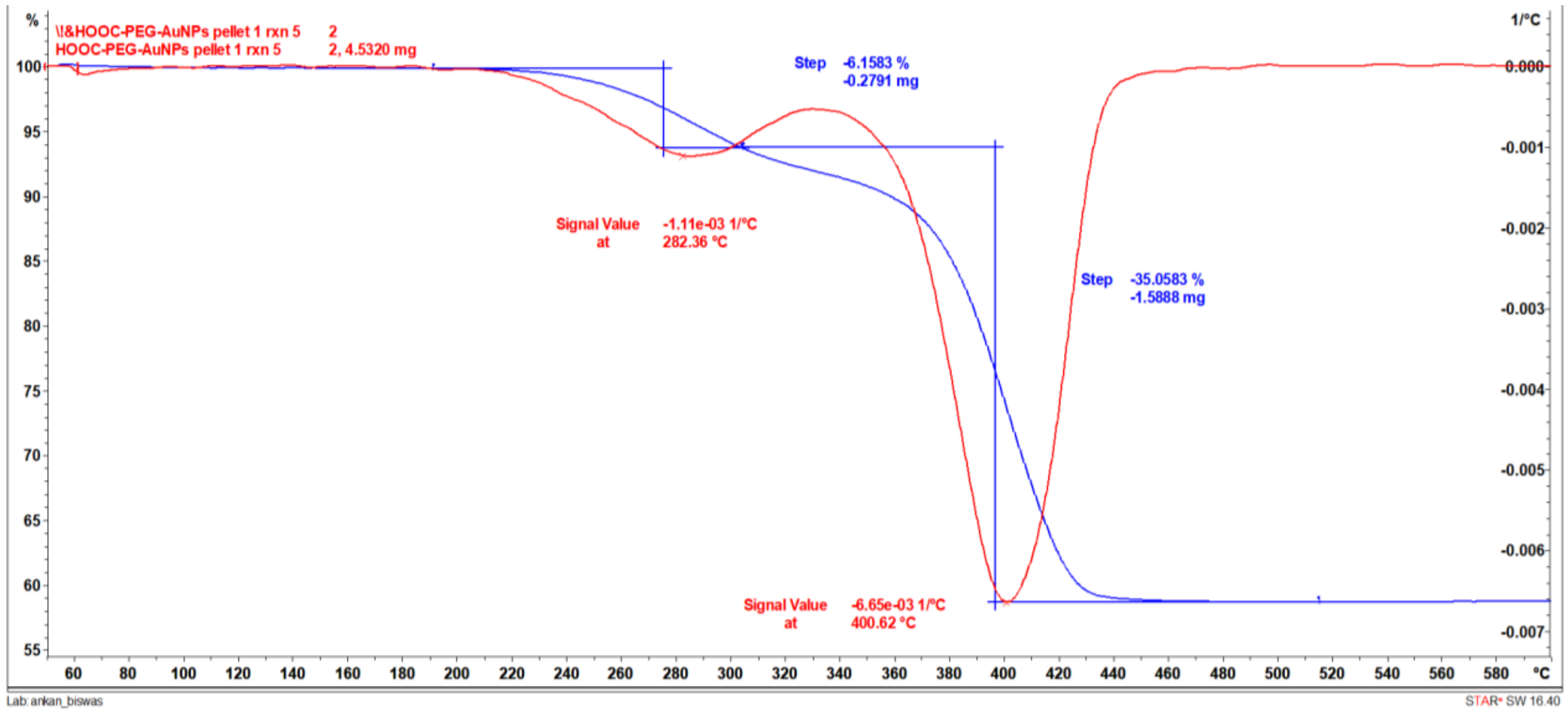

HOOC-PEG-AuNPs pellet #2

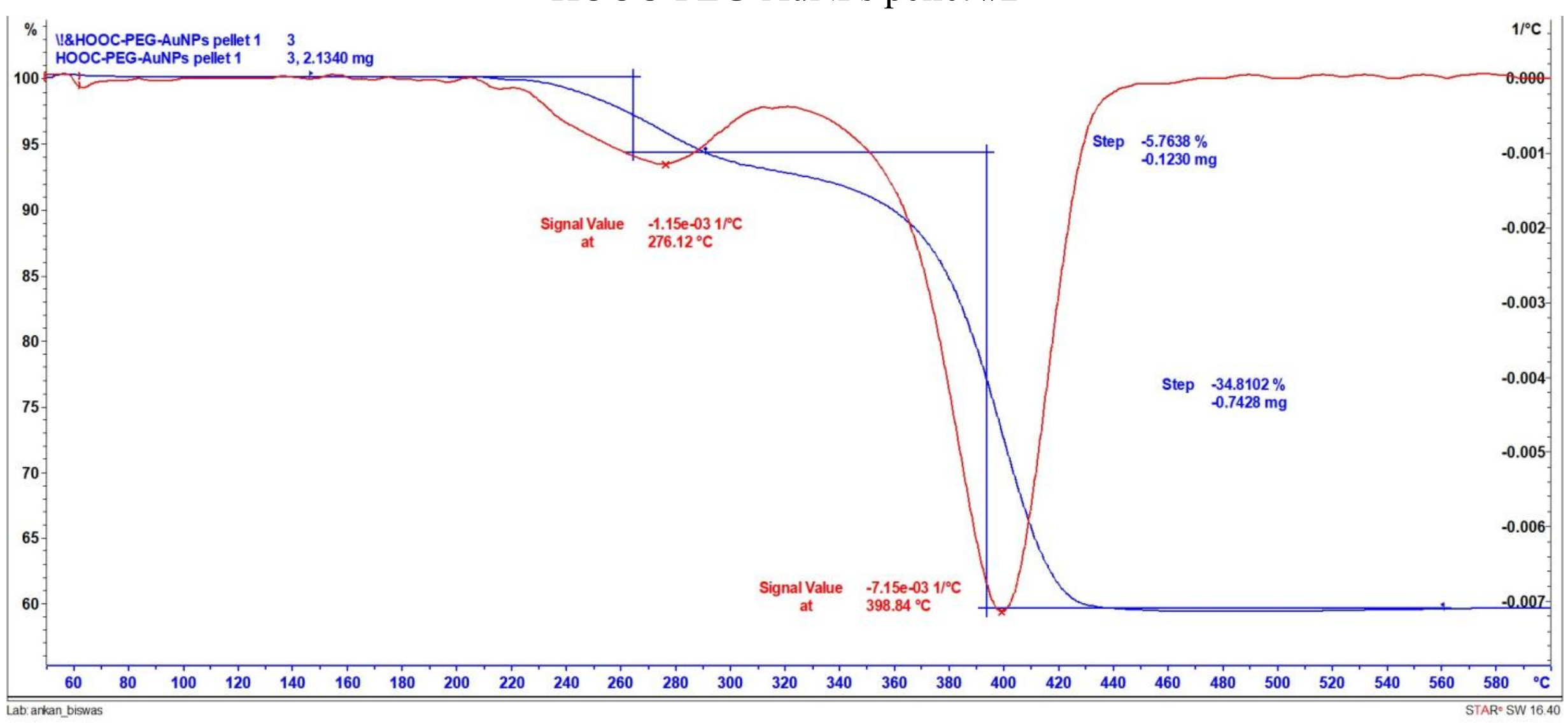

HOOC-PEG-AuNPs pellet #3

Figure S10. TGA graphs of **purified HOOC-PEG-AuNPs**.

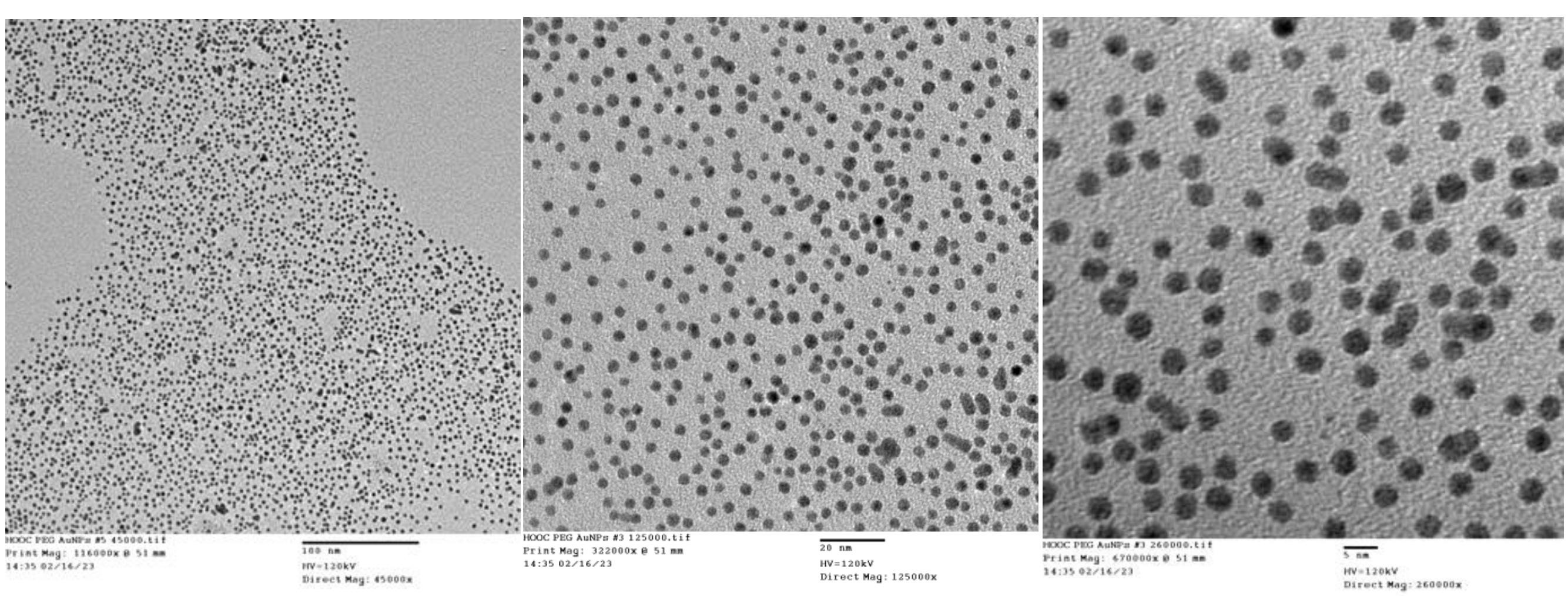

Figure S11. TEM images of **3.2** ± 0.4 **nm** HOOC-PEG-AuNPs at 0.5 mg/mL in water. The examined magnitudes (left to right) were 45000x, 125000x and 260000x.

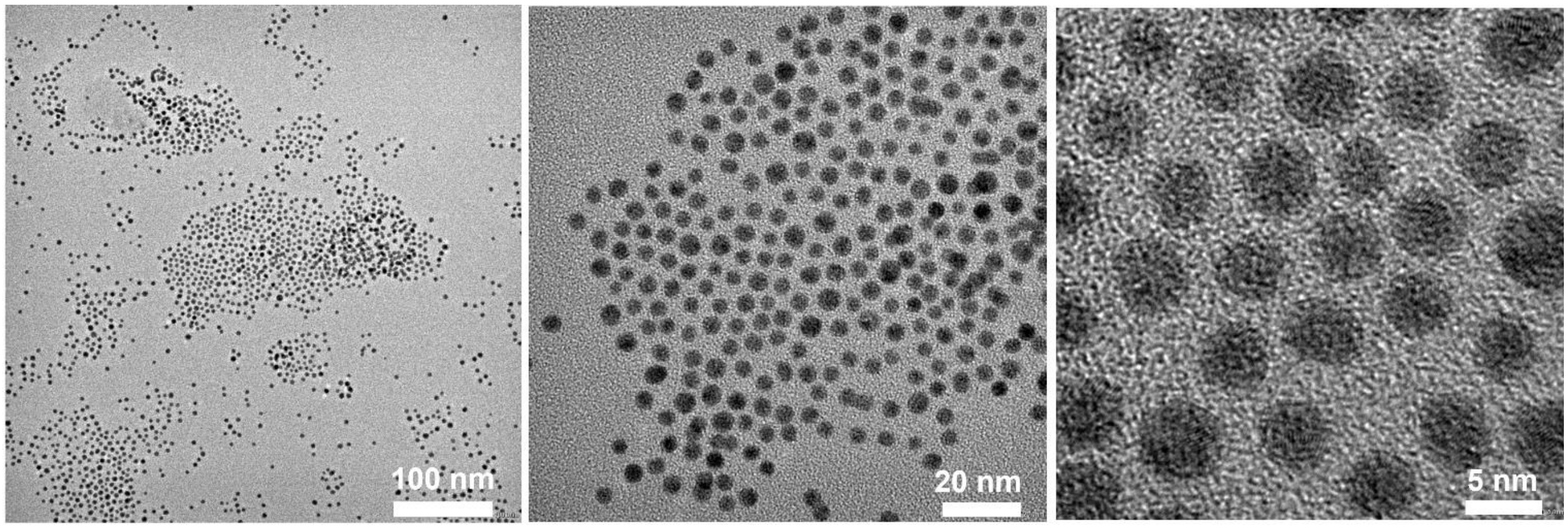

Figure S12. TEM images of **4.2** ± 0.6 **nm** *GAT1508*-PEG-AuNPs at 0.5 mg/mL in water at different magnifications (left to right).

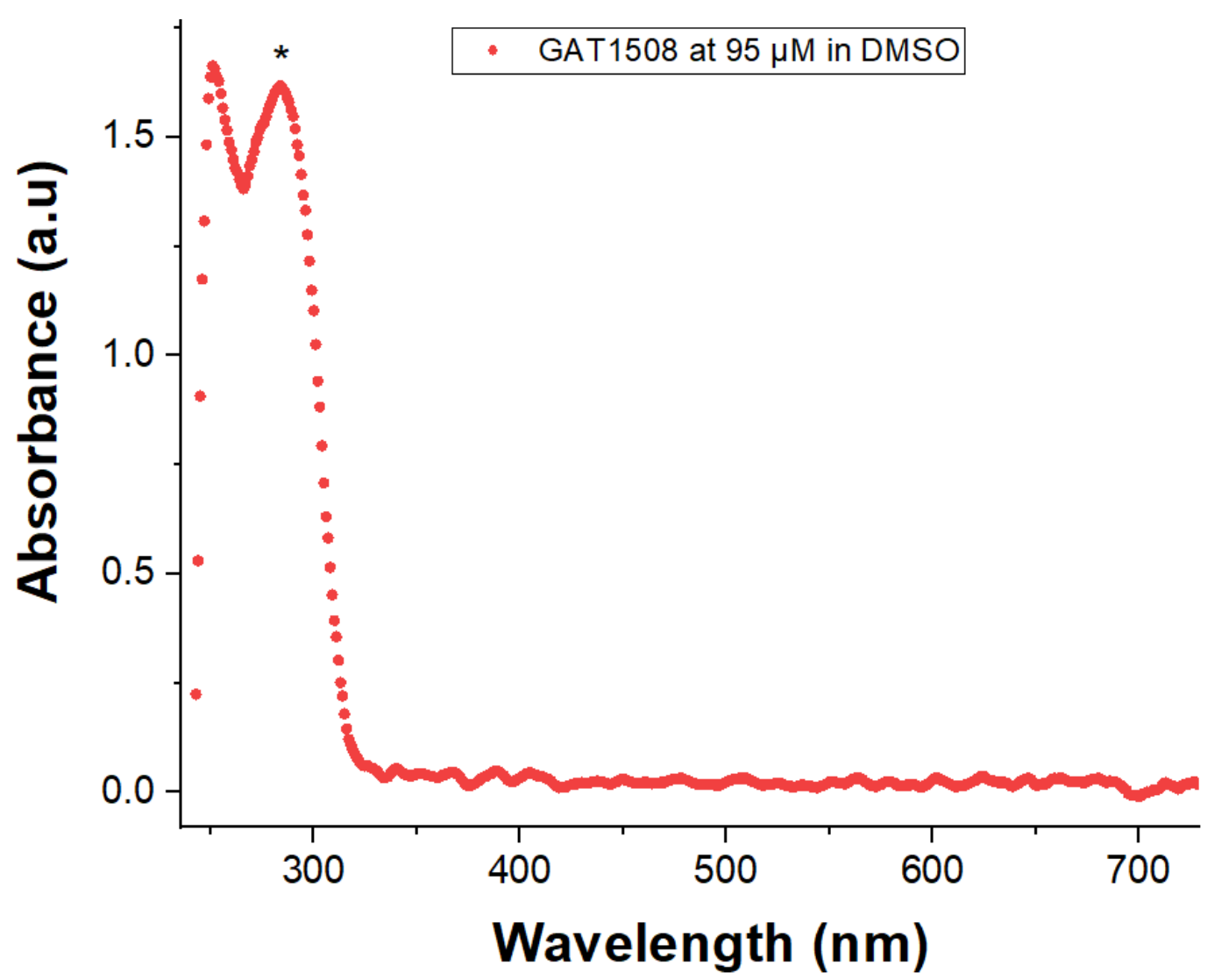

Figure S13. UV-Vis of *GAT1508* at 95 µM in DMSO.

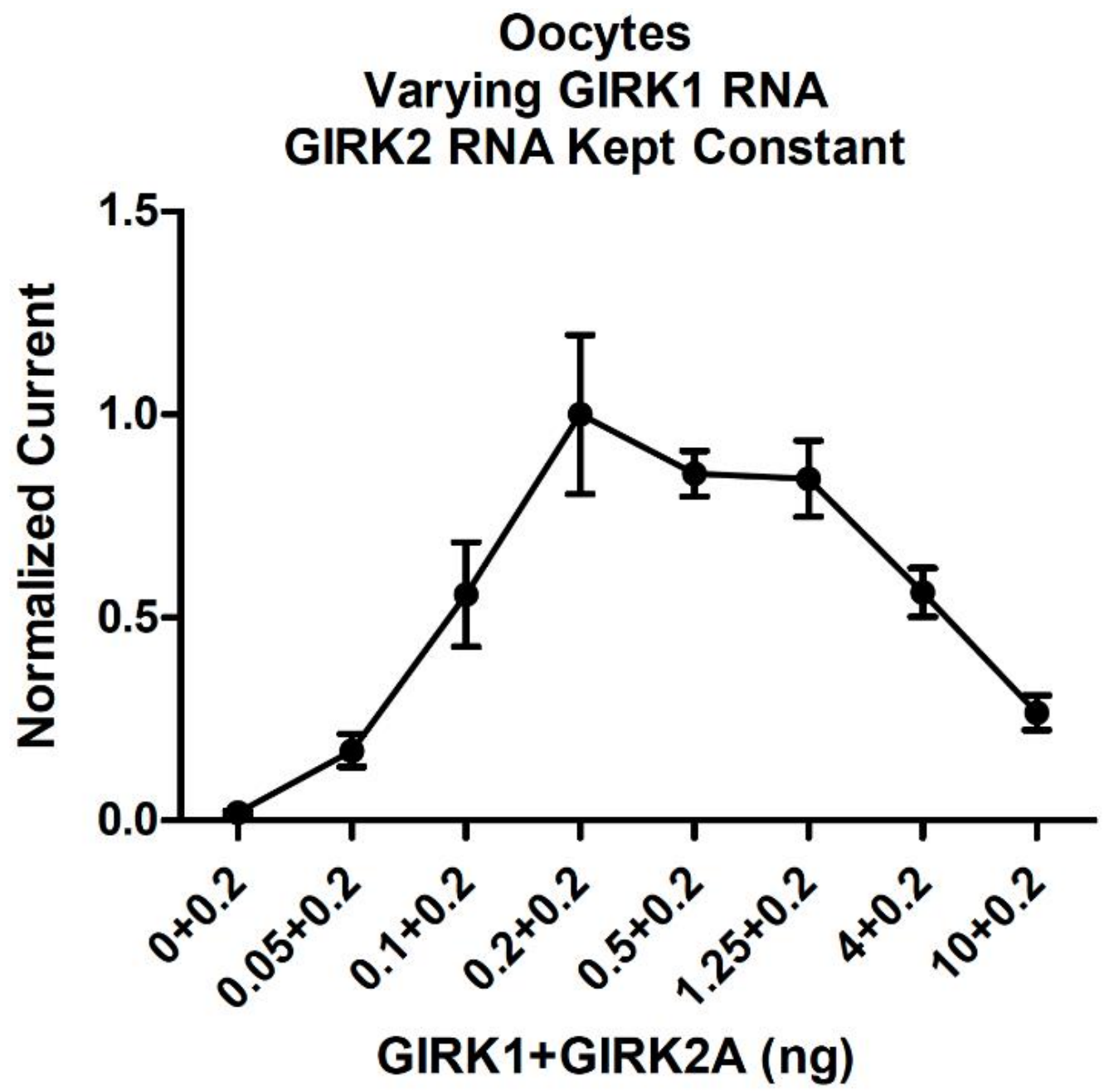

Figure S14. GIRK1/GIRK2 injected RNA ratios from 0.25:1 up to 50:1 can lead to the formation of transmembrane GIRK1/2 tetrameric units enabling ion channel-opening and flow of electric current.

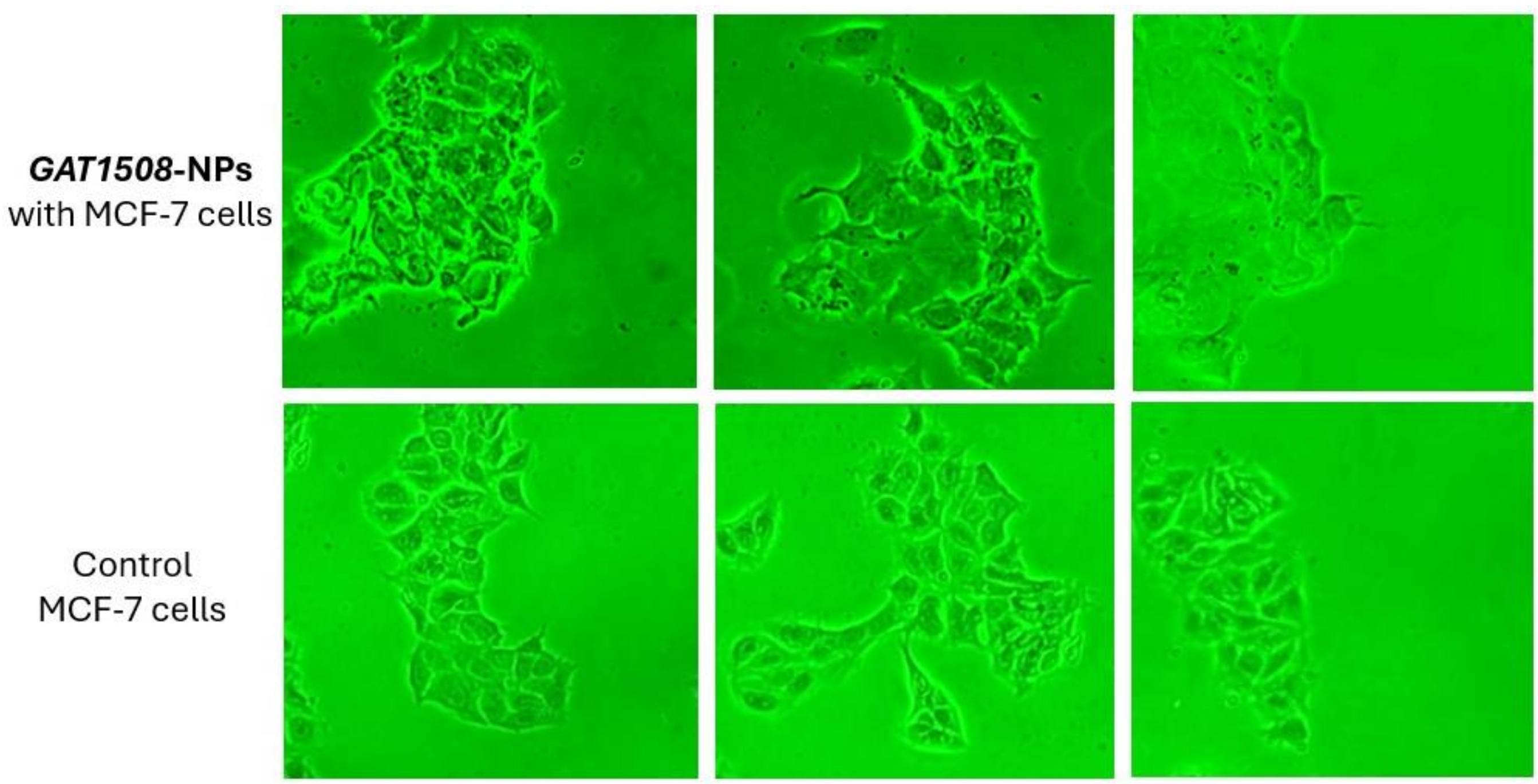

Figure S15. Optical microscope images of MCF-7 cells in the presence of GAT1508-coated NPs obtained at a different (basic) optical microscope with a cell phone camera.

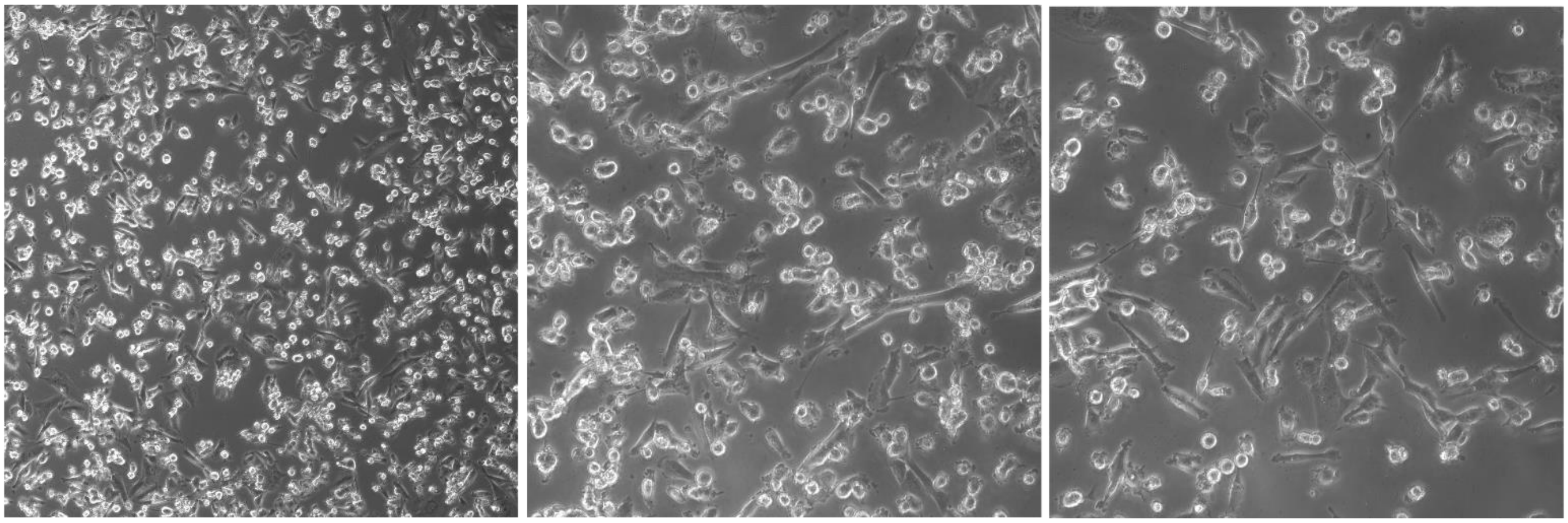

Figure S16. Optical microscope images of triple (-) MDA-MB-231 cells that do not express GIRK1/2 after incubation with *GAT1508*-coated NPs showing absence of interaction.

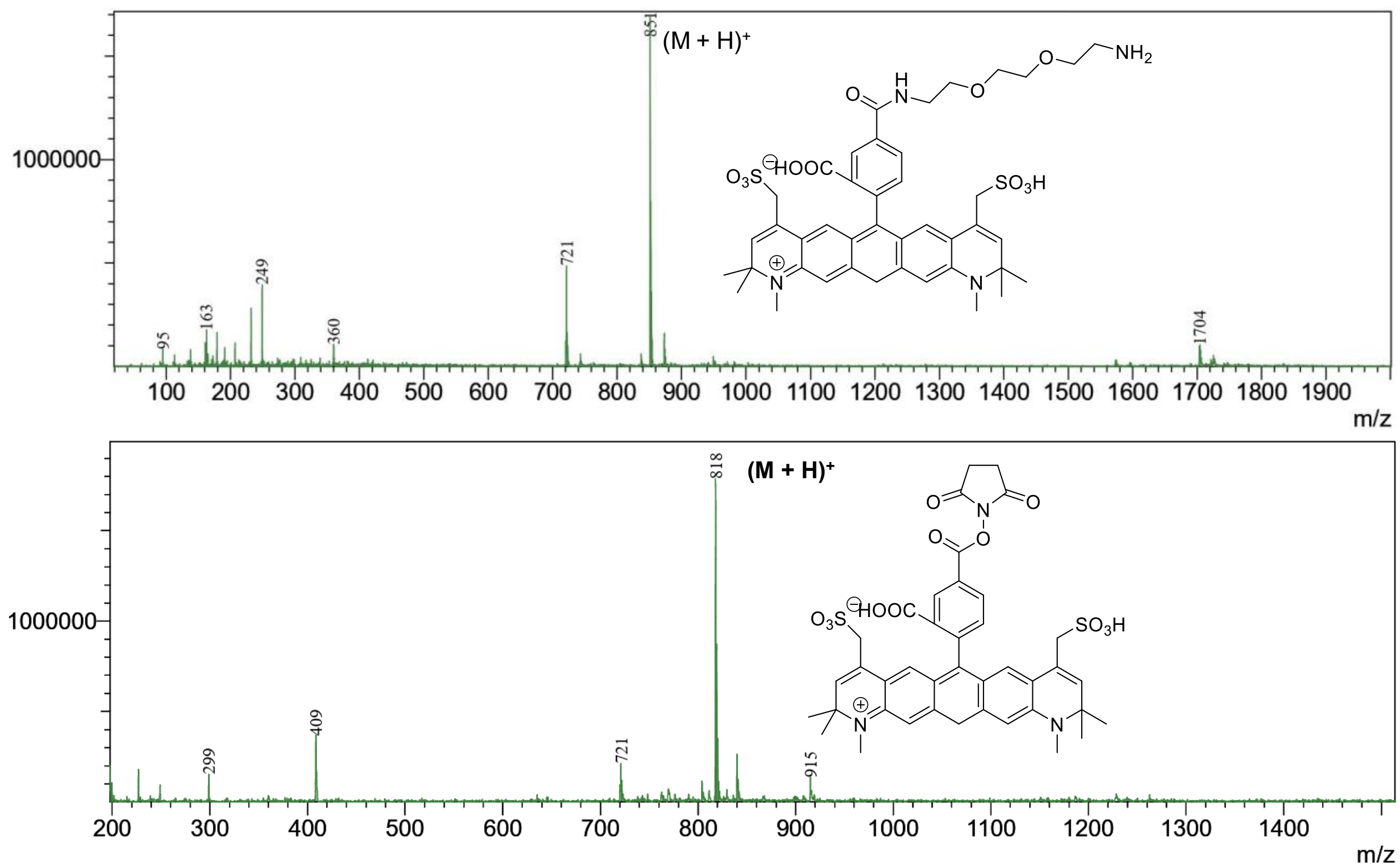

Figure S17. LC-MS spectra of (a) Alexa Fluor 570-EG$_2$-NH$_2$ (MW = 851 g/mol) and (b) starting material Alexa Fluor 594 NHS ester (MW = 818 g/mol).
